# Supplementary material for: Nostoc edaphicum CCNP1411 from the Baltic Sea—A New Producer of Nostocyclopeptides
Source: Mar Drugs. 2020 Aug 26;18(9):442. doi: 10.3390/md18090442 (PMC7551626; doi:10.3390/md18090442)
Supplement: Supplementary file 1 [file marinedrugs-18-00442-s001.pdf]

Supplementary Material: *Nostoc edaphicum* CCNP1411 from the Baltic Sea – a new producer of nostocyclopeptides

Anna Fidor [1], Michał Grabski [2], Jan Gawor [3], Robert Gromadka [3], Grzegorz Węgrzyn [2], Hanna Mazur-Marzec [1*]

[1] Division of Marine Biotechnology, Faculty of Oceanography and Geography, University of Gdansk, Marszałka J. Piłsudskiego 46, PL-81378 Gdynia, Poland; anna.fidor@phdstud.ug.edu.pl (A.F.);
[2] Department of Molecular Biology, University of Gdansk, Wita Stwosza 59, 80-308 Gdansk, Poland; grzegorz.wegrzyn@biol.ug.edu.pl (G.W.); michal.grabski@phdstud.ug.edu.pl (M.G.);
[3] Institute of Biochemistry and Biophysics, Polish Academy of Sciences, DNA Sequencing and Oligonucleotide Synthesis Laboratory, Warsaw, Poland; gaworj@ibb.waw.pl (J.G.); robert@ibb.waw.pl (R.G.)
* Correspondence: hanna.mazur-marzec@ug.edu.pl; Tel.: +48 -58-523-66-21; +609-419-132 (H.M.M.)

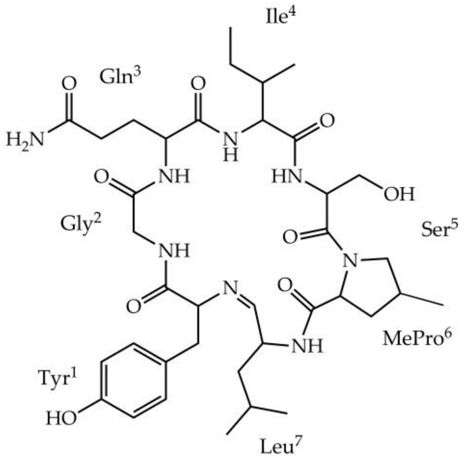

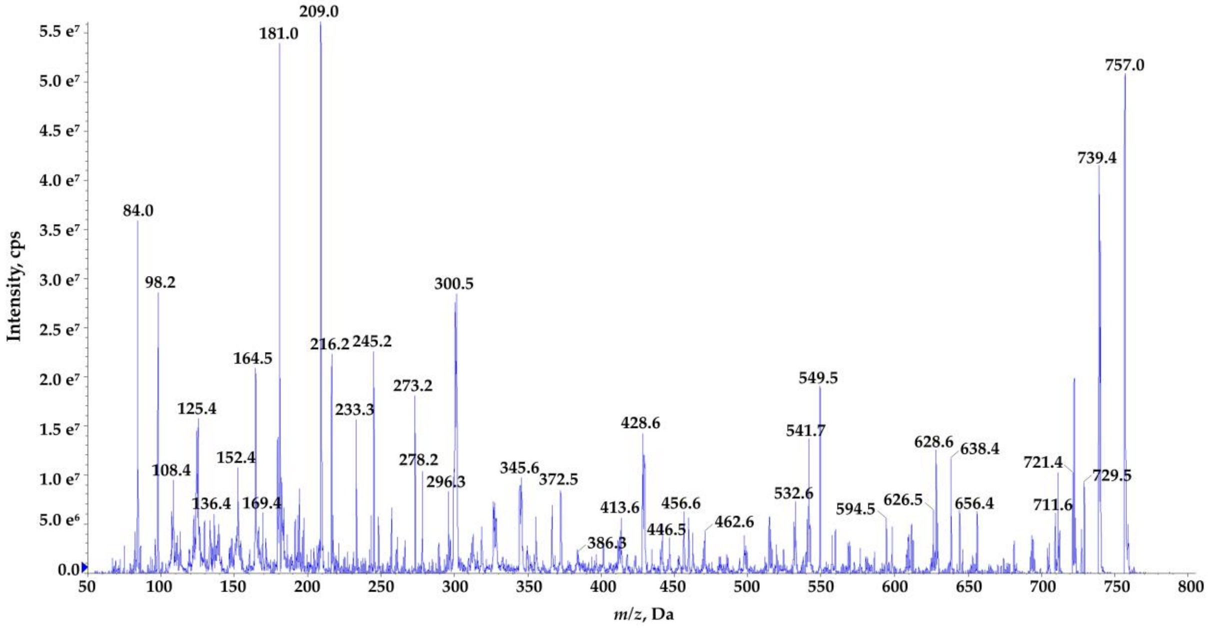

Figure S1: Structure and enhanced product ion mass spectrum of the cyclic nostocyclopeptide **Ncp-A1** cyclo[Tyr+Gly+Gln+Ile+Ser+MePro+Leu] identified based on the following fragment ions: ***m/z* 757** [M+H]; 739 [M+H – H_2O]; 729 [M+H – CO]; 721 [M+H – $2H_2O$]; 628 [M+H – MePro – H_2O], 626 [M+H – Ile – H_2O]; 594 [M+H – Tyr]; 549 [Tyr+Gly+Gln+Ile+Ser+H]; 541 [M+H – (Ser+MePro) – H_2O], 446 [M+H – (Ile+Ser+MePro)]; 428 [M+H – (Ile+Ser+MePro) – H_2O]; 386 [Gly+Gln+Ile+Ser+H]; 372 [MePro+Leu+Tyr+H]; 300 [Leu+Tyr+Gly+H – H_2O]; 209 [MePro+Leu+H]; 181 [MePro+Leu+H – CO]; 86 – Ile/Leu immonium; 84 MePro immonium.

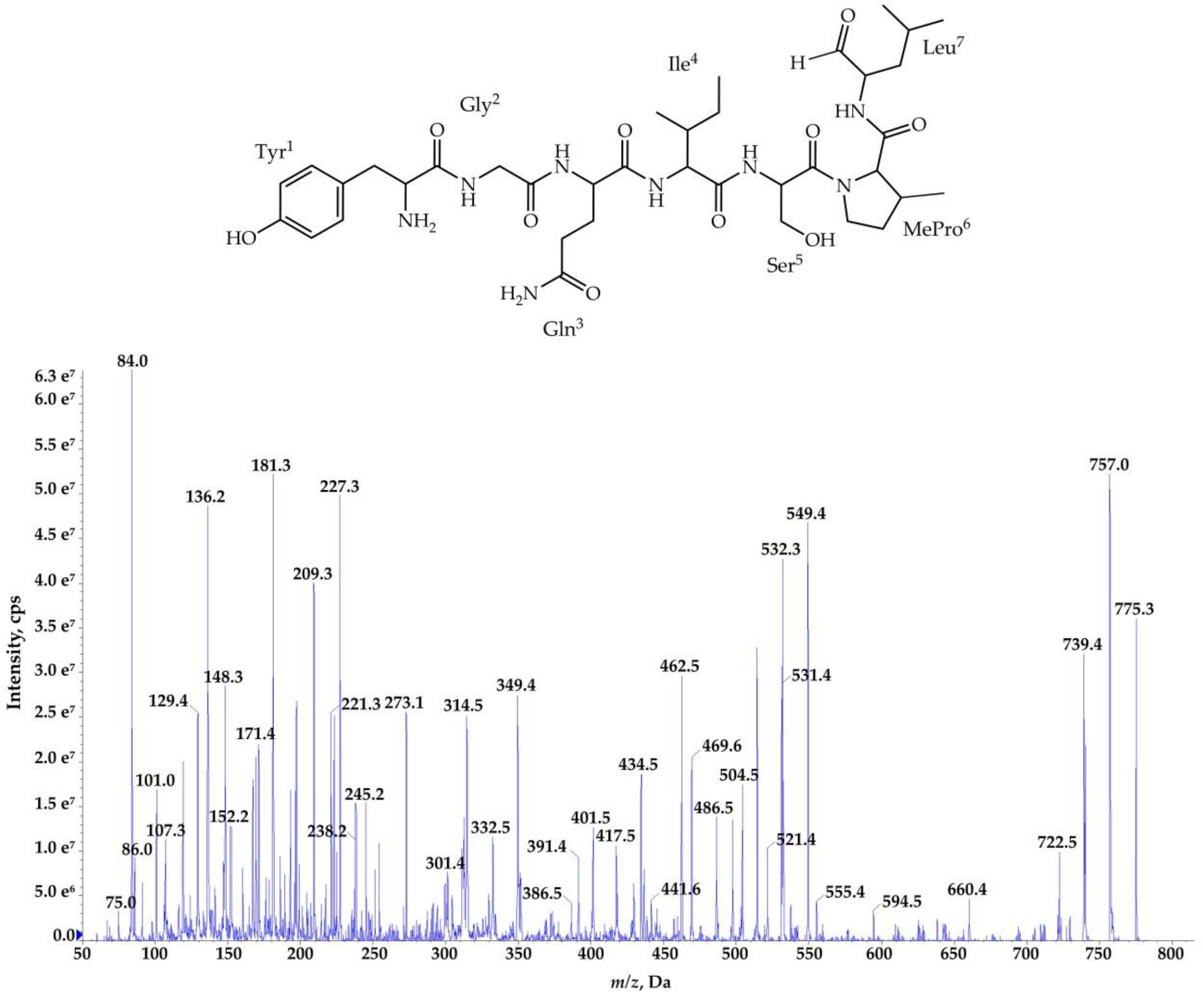

Figure S2: Structure and enhanced product ion mass spectrum of the linear peptide aldehyde **Ncp-A1-L** (linear aldehyde of **Ncp-A1**) Tyr+Gly+Gln+Ile+Ser+MePro+Leu identified based on the following fragment ions**: *m/z* 775** [M+H]; 757 [M+H – H_2O]; 739 [M+H – $2H_2O$]; 660 [M+H – Leu]; 549 [Tyr+Gly+Gln+Ile+Ser+H]; 531 [Tyr+Gly+Gln+Ile+Ser+H – H_2O]; 521 [Tyr+Gly+Gln+Ile+Ser+H – CO]; 532 [M+H – (MePro+Leu) – H_2O]; 462 [Tyr+Gly+Gln+Ile+H]; 434 [Tyr+Gly+Gln+Ile+H – CO]; 386 [Gly+Gln+Ile+Ser+H]; 349 [Tyr+Gly+Gln+H]; 301 [Gln+Ile+Ser+H – CO]; 227 [MePro+Leu+H]; 221 [Tyr+Gly+H]; 209 [MePro+Leu+H – H_2O]; 181 [MePro+Ile+H – H_2O – CO]; 148 [Tyr – NH_2]; 136 Tyr immonium; 86 – Ile/Leu immonium; 84, 101 (immonium) ,129 Gln; 84 MePro immonium.

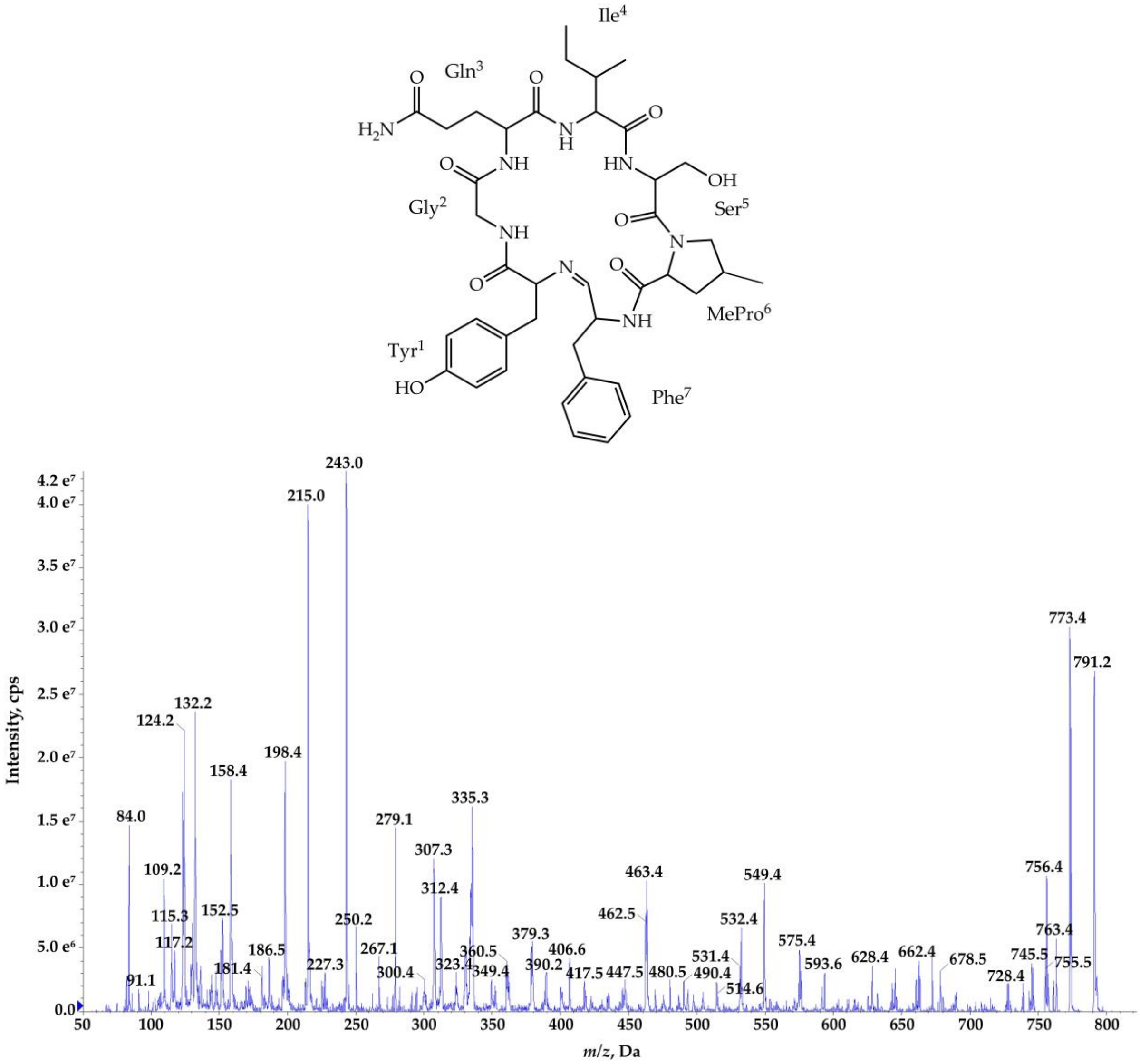

Figure S3: Structure and enhanced product ion mass spectrum of the cyclic nostocyclopeptide **Ncp-A2** cyclo[Tyr+Gly+Gln+Ile+Ser+MePro+Phe] identified based on the following fragment ions: ***m/z* 791** [M+H]; 773 [M+H – H_2O]; 763 [M+H – CO]; 755 [M+H – $2H_2O$]; 745 [M+H – CO – H_2O]; 678 [M+H – Ile]; 628 [M+H – Tyr]; 593 [M+H – (Ser+MePro)]; 549 [Tyr+Gly+Gln+Ile+Ser+H]; 531 [Tyr+Gly+Gln+Ile+Ser+H – H_2O]; 480 [M+H – (Ile+Ser+MePro)]; 462 [Tyr+Gly+Gln+Ile+H]; 406 [MePro+Phe+Tyr+H]; 379 [MePro+Phe+Tyr+H – CO]; 349 [Tyr+Gly+Gln+H]; 335 [Phe+Tyr+Gly+H – H_2O]; 312 [Ile+Ser+MePro+H]; 307 [Phe+Tyr+Gly+H – H_2O – CO]; 243 [MePro+Phe+H]; 215 [MePro+Phe+H – CO]; 158 [Gly+Gln+H – CO]; 132 Phe; 84 MePro immonium.

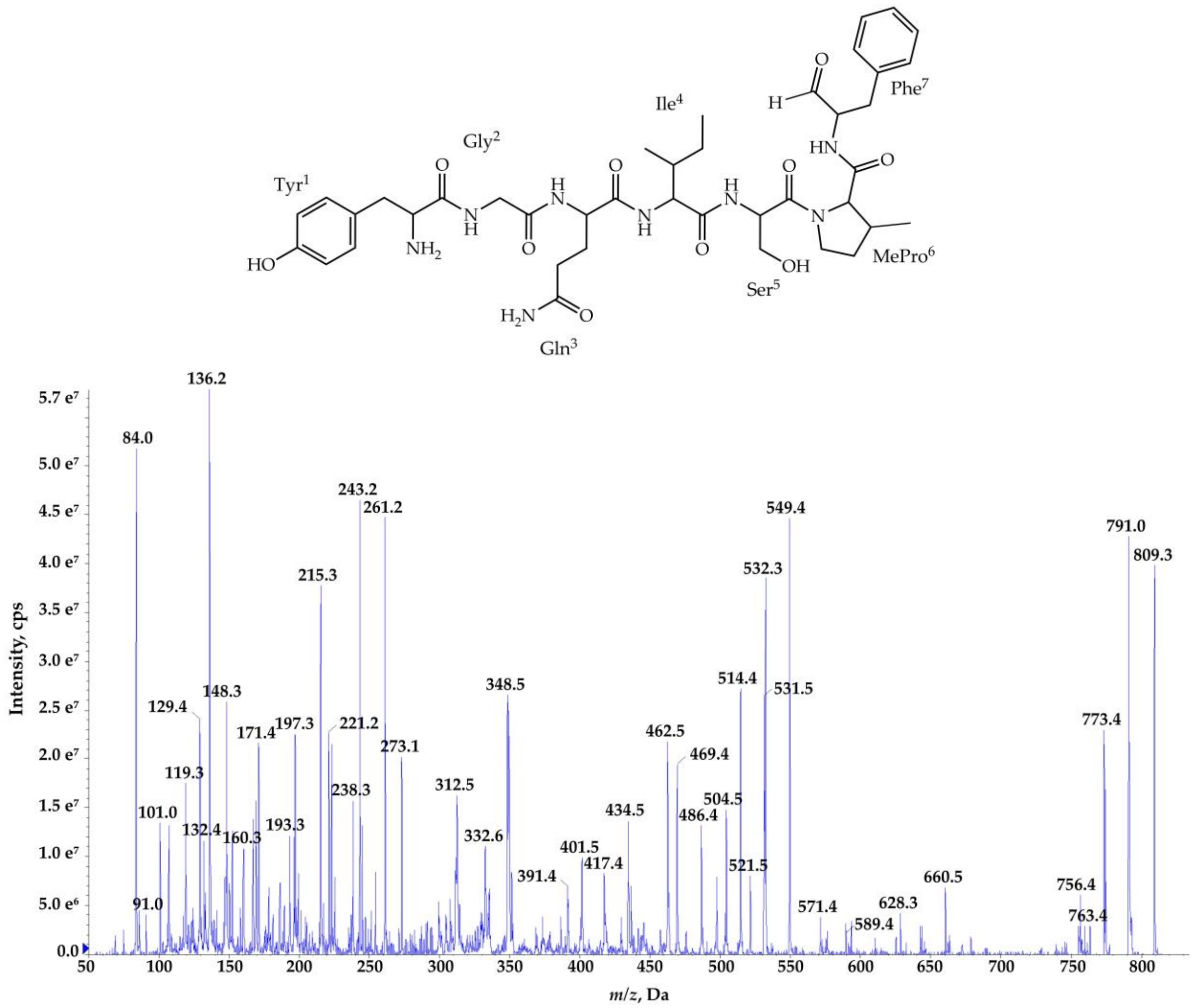

Figure S4: Structure and enhanced product ion mass spectrum of the linear nostocyclopeptide aldehyde **Ncp-A2-L** (linear aldehyde of **Ncp-A2)** Tyr+Gly+Gln+Ile+Ser+MePro+Phe identified based on the following fragment ions: ***m/z* 809** [M+H]; 791 [M+H – H_2O]; 773 [M+H – $2H_2O$]; 763 [M+H – CO – H_2O]; 660 [M+H – Phe]; 628 [M+H – Tyr – H_2O]; 549 [Tyr+Gly+Gln+Ile+Ser+H]; 531 [M+H – (MePro+Phe) – H_2O]; 462 [Tyr+Gly+Gln+Ile+H]; 434 [Tyr+Gly+Gln+Ile+H – CO]; 312 [Ile+Ser+MePro+H]; 261 [MePro+Phe+H]; 243 [MePro+Phe+H – H_2O]; 221 [Tyr+Gly+H], 193 [Tyr+Gly+ H – CO]; 148 [Tyr – NH_2]; 136 Tyr immonium; 84, 101 (immonium), 129 Gln; 84 MePro immonium.

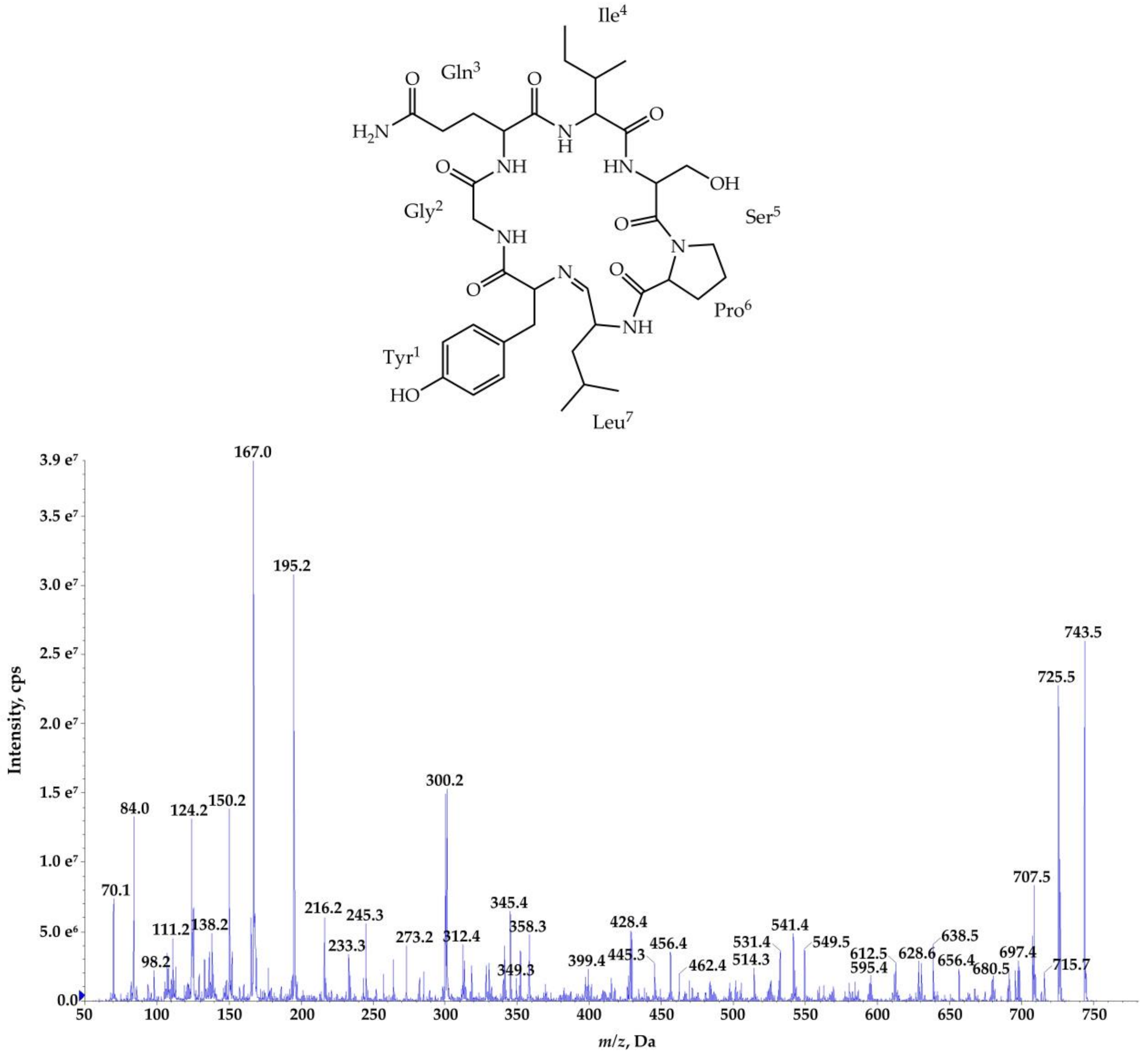

Figure S5: Proposed structure and enhanced product ion mass spectrum of cyclic nostocyclopeptide **Ncp-E2** cyclo[Tyr+Gly+Gln+Ile+Ser+Pro+Leu] characterized based on the following fragment ions: ***m/z* 743** [M+H]; 725 [M+H – H_2O]; 715 [M+H – CO]; 707 [M+H – $2H_2O$]; 697 [M+H – H_2O – CO]; 656 [M+H – Ser]; 638 [M+H – Ser – H_2O]; 628 [M+H – Ser – CO]; 612 [M+H – Ile – H_2O]; 549 [Tyr+Gly+Gln+Ile+Ser+H]; 541 [M+H – (Ser+Pro) – H_2O]; 531 [Tyr+Gly+Gln+Ile+Ser+H – H_2O]; 428 [M+H – (Ile+Ser+Pro) – H_2O]; 349 [Tyr+Gly+Gln+H]; 300 [Leu+Tyr+Gly+H – H_2O]; 195 [Pro+Leu+H]; 167 [Pro+Leu+H – CO]; 84 Gln; 70 Pro immonium.

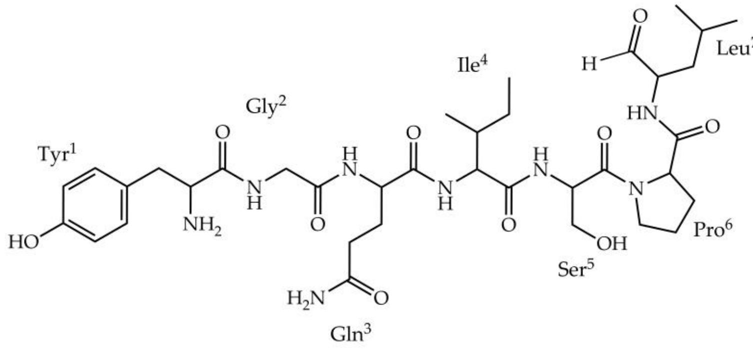

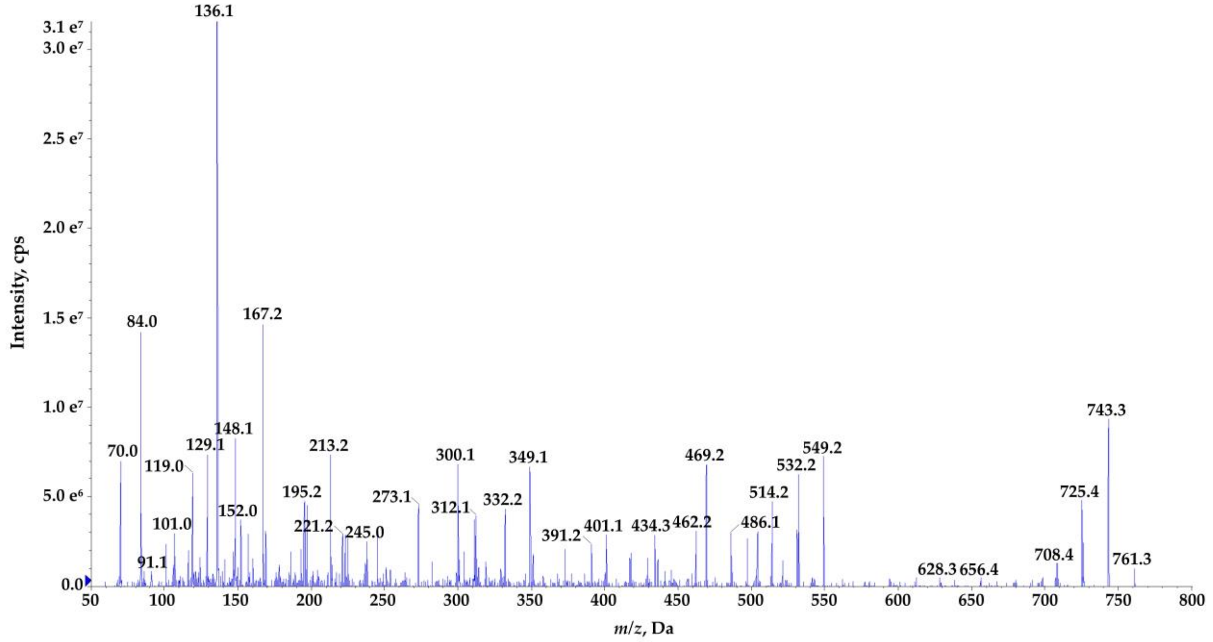

Figure S6: Proposed structure and enhanced product ion mass spectrum of the linear nostocyclopeptide aldehyde **Ncp-E2-L** (linear aldehyde of **Ncp-E2**) with general structure Tyr+Gly+Gln+Ile+Ser+Pro+Leu characterized based on the following fragment ions: ***m/z* 761** [M+H]; 743 [M+H – H_2O]; 725 [M+H – $2H_2O$]; 549 [Tyr+Gly+Gln+Ile+Ser+H]; 532 [Tyr+Gly+Gln+Ile+Ser+H – H_2O]; 462 [Tyr+Gly+Gln+Ile+H]; 349 [Tyr+Gly+Gln+H]; 434 [Tyr+Gly+Gln+Ile+H – CO]; 300 [Ser+Pro+Leu+H]; 221 [Tyr+Gly+H]; 213 [Pro+Leu+H]; 195 [Pro+Leu+H – H_2O]; 148 [Tyr – NH_2]; 136 Tyr immonium; 84, 101 (immonium), 129 Gln; 70 Pro immonium.

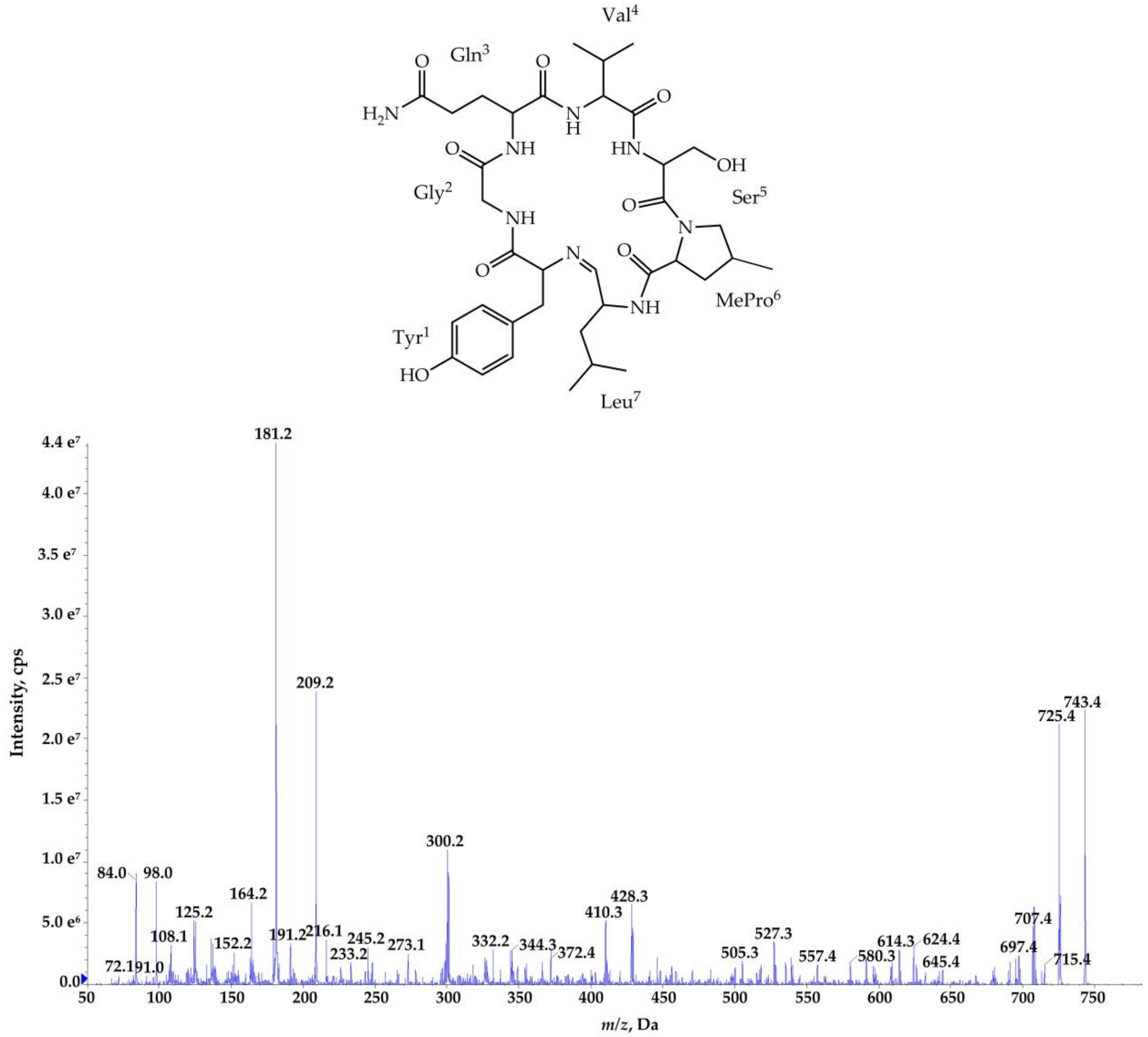

Figure S7: Proposed structure and enhanced product ion mass spectrum of cyclic nostocyclopeptide **Ncp-E3** cyclo[Tyr+Gly+Gln+Val+Ser+MePro+Leu] characterized based on the following fragment ions: ***m/z* 743** [M+H]; 725 [M+H – H_2O]; 715 [M+H – CO]; 707 [M+H – 2H_2O]; 697 [M+H – H_2O – CO]; 645 [M+H – Val]; 580 [M+H – Tyr]; 527 [M+H – (Ser+MePro) – H_2O]; 428 [M+H – (Val+Ser+MePro) – H_2O]; 410 [M+H – (Val+Ser+MePro) – 2H_2O]; 372 [Gly+Gln+Val+Ser+H] ; 344 [Gly+Gln+Val+Ser+H – CO]; 300 [Leu+Tyr+Gly+H – H_2O]; 233 [Leu+Tyr+H – CO]; 209 [MePro+Leu+H]; 181 [MePro - Leu+H – CO]; 84 MePro immonium; 72 Val immonium.

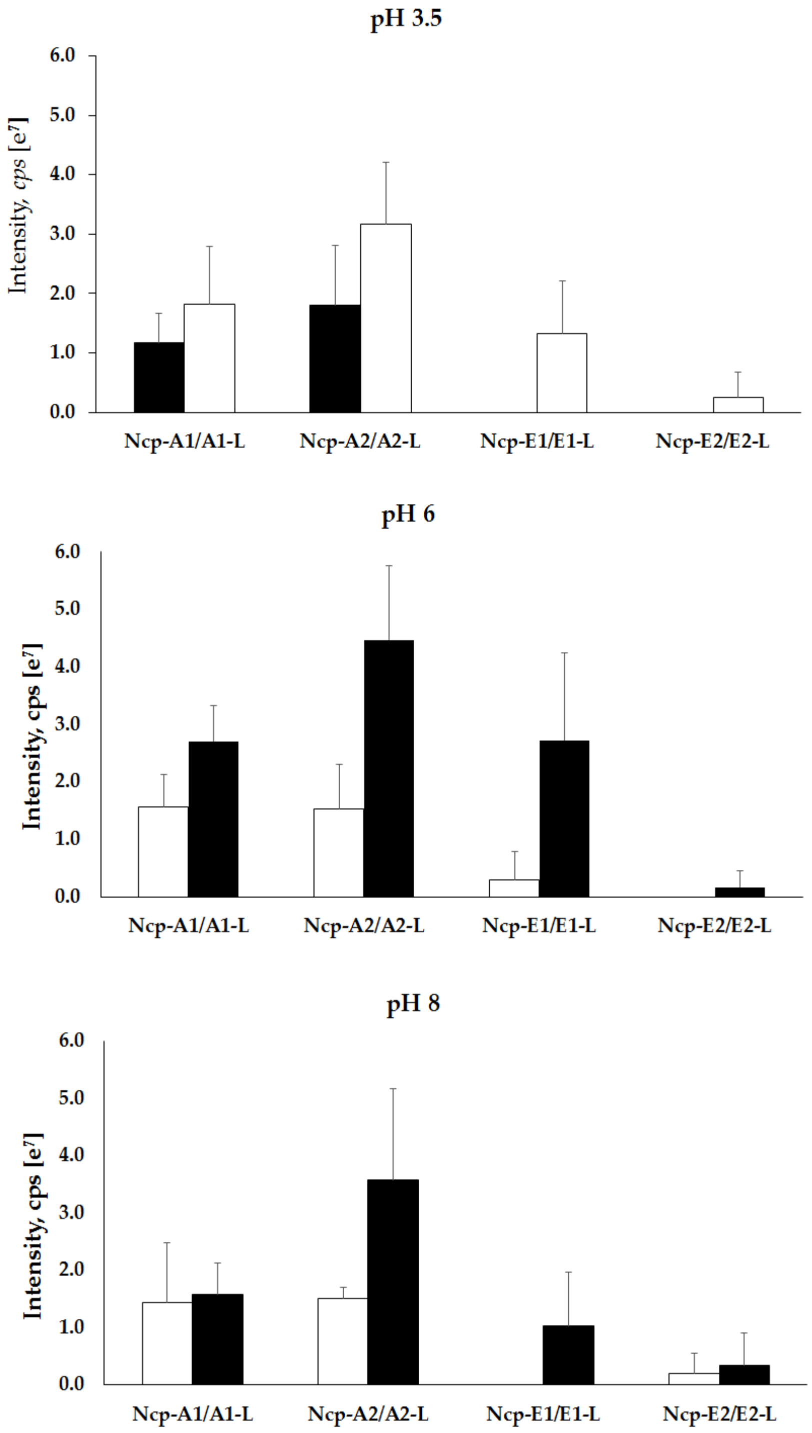

Figure S8: Relative cell contents of nostocyclopeptides extracted from 10 mg of lyophilized biomass of *N. edaphicum* CCNP1411 with 20% MeOH in different pH (3.5, 6 and 8).
